# The Bolingbroke Hospital

**Published:** 1906-03-17

**Authors:** 


### THE BOLINGBROKE HOSPITAL.

HANDSOME CONTRIBUTION TO THE BUILDING FUND.

At the annual general meeting of the proprietors of Price's Patent Candle Company, Limited, held at Cannon Street Hotel on Friday, March 9, the report submitted by the directors contained the following paragraph:—

"In 1897 the shareholders, on the recommendation of the directors, authorised a donation of five hundred guineas to the Building and Endowment Fund of the Bolingbroke Hospital. The hospital, which is situated in the neighbourhood of the Battersea works, is now being enlarged to meet the necessities of the district, and to bring it up to date with modern requirements. The directors recommend that the company should contribute a further five hundred guineas in augmentation of the Building Fund."

In the course of his speech, the Chairman (Sir Peter Spokes) said: The contribution to the Building Fund of the Bolingbroke Hospital, for which the directors venture to ask your sanction may be regarded as maintaining the efficiency of an institution which is at all times available for a community of which our own workpeople at the Battersea works form no inconsiderable part. The company has been so closely associated with the hospital from its inception and foundation, through the late managing director, Mr. Calderwood, that it seems only right to assist in extending it and in bringing it up to date in all modern requirements. The population of the district immediately surrounding the hospital has practically doubled during the last twenty-five years and approximates to half a million, or a twelfth part of the population of the Metropolitan area. A full staff of surgeons and physicians has been appointed, so that it partakes of the character of a general hospital with in-patient and out-patient departments, and has received the countenance and support of the committee of King Edward's Hospital Fund. The directors are unanimously of opinion that Price's, as the largest employers of labour in the neighbourhood, should not withhold their further help at this juncture of its history. They, therefore, confidently commend it to the generosity of the shareholders, and will, before the meeting separates, submit a resolution to give effect to this recommendation.

At a later stage of the meeting the Chairman proposed a formal resolution approving a contribution by the company of a further 500 guineas in augmentation of the Building Fund of the hospital; and the Vice-Chairman, in seconding this, mentioned that one of the company's workmen was at the present time an in-patient.

Subsequently, in proposing a vote of thanks to the Chairman and directors, which was cordially adopted, Mr. A. Glegg, as Mayor of Wandsworth, expressed pleasure at the interest which this company showed in a practical way in a hospital within his own district, and said it was very gratifying to see the company pioneering these philanthropic movements.

